# Supplementary material for: Detection and validation of QTLs for flowering time in morning glory
Source: Breed Sci. 2025 Oct 15;75(5):339–48. doi: 10.1270/jsbbs.24067 (PMC13129579; doi:10.1270/jsbbs.24067)
Supplement: Supplementary file 1 — Supplemental Figures [file 75_339_s1.pdf]

Katsuyama et al./Breeding Science
doi: 10.1270/jsbbs.24067

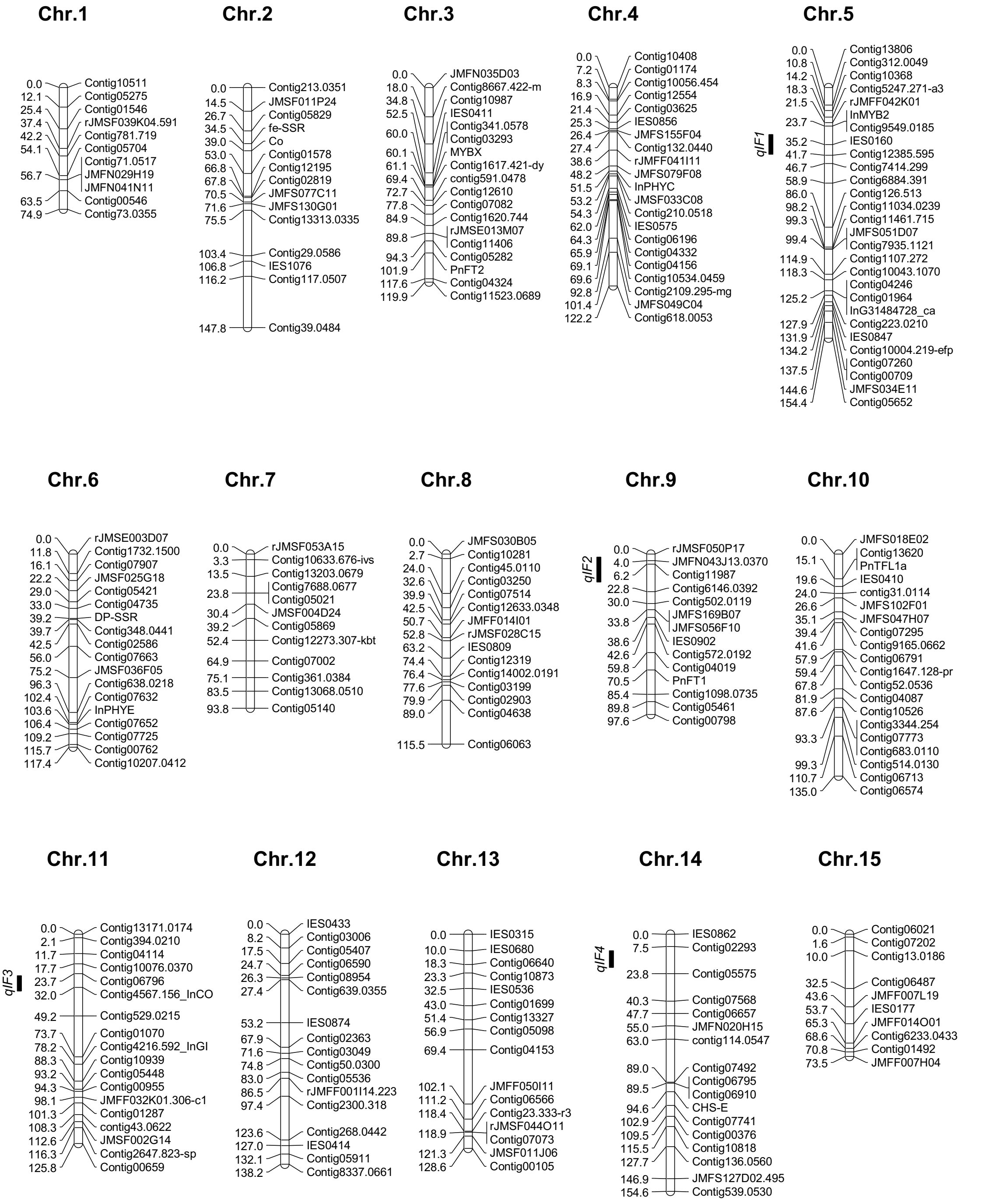

Supplemental Fig. 1. Genetic linkage map of F_2 population derived from the cross between Q65 × TKS and cultivated in 2011. The marker distance (cM) is presented on the left side of each chromosome. Marker names are shown on the right side. Vertical black bars show QTL regions for flowering days with 99% creditable intervals.

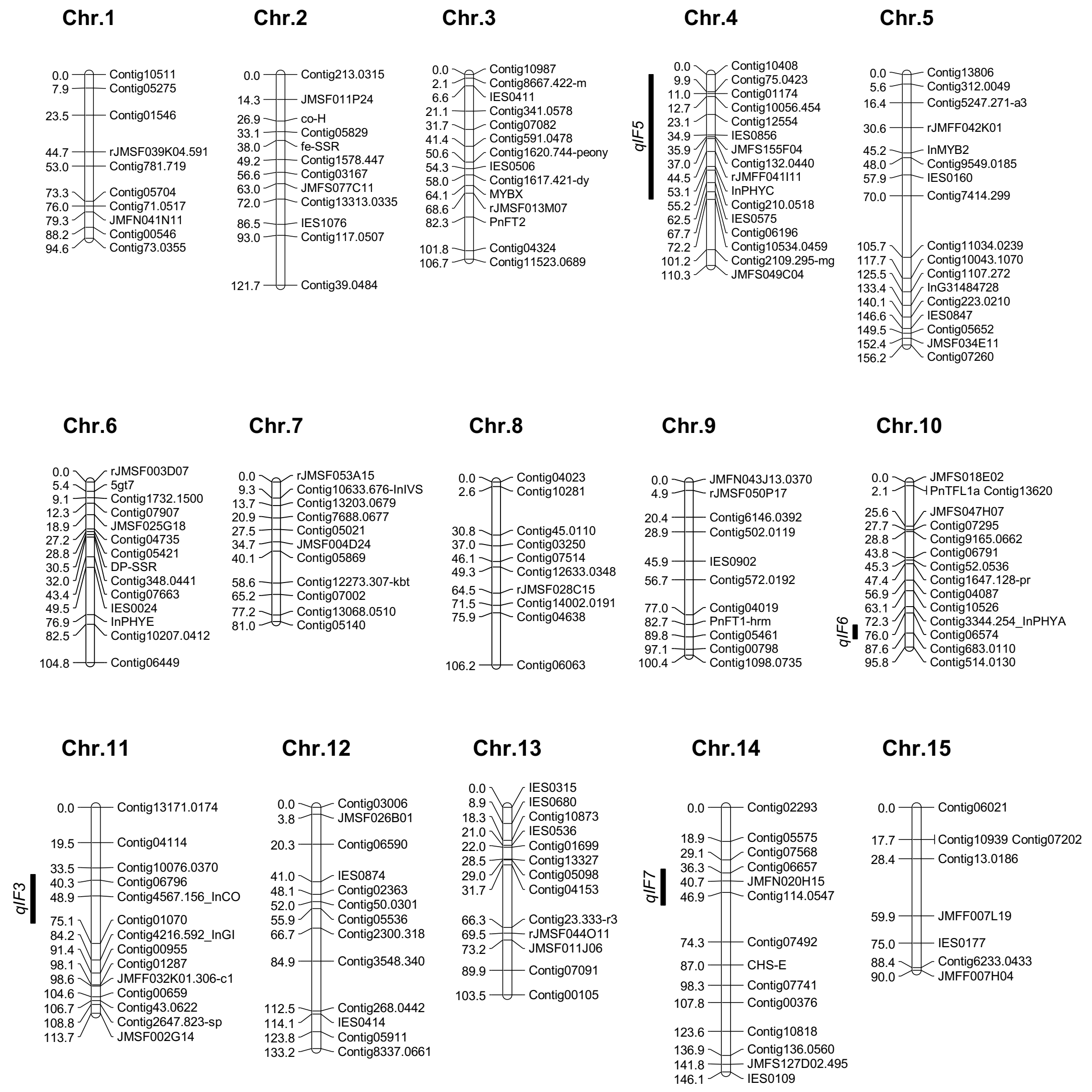

Supplemental Fig. 2. Genetic linkage map of F_2 population derived from the cross between Q65 × TKS and cultivated in 2012. The marker distance (cM) is presented on the left side of each chromosome. The marker names are shown on the right side. Vertical black bars show QTLs region for flowering days with 99% creditable intervals.

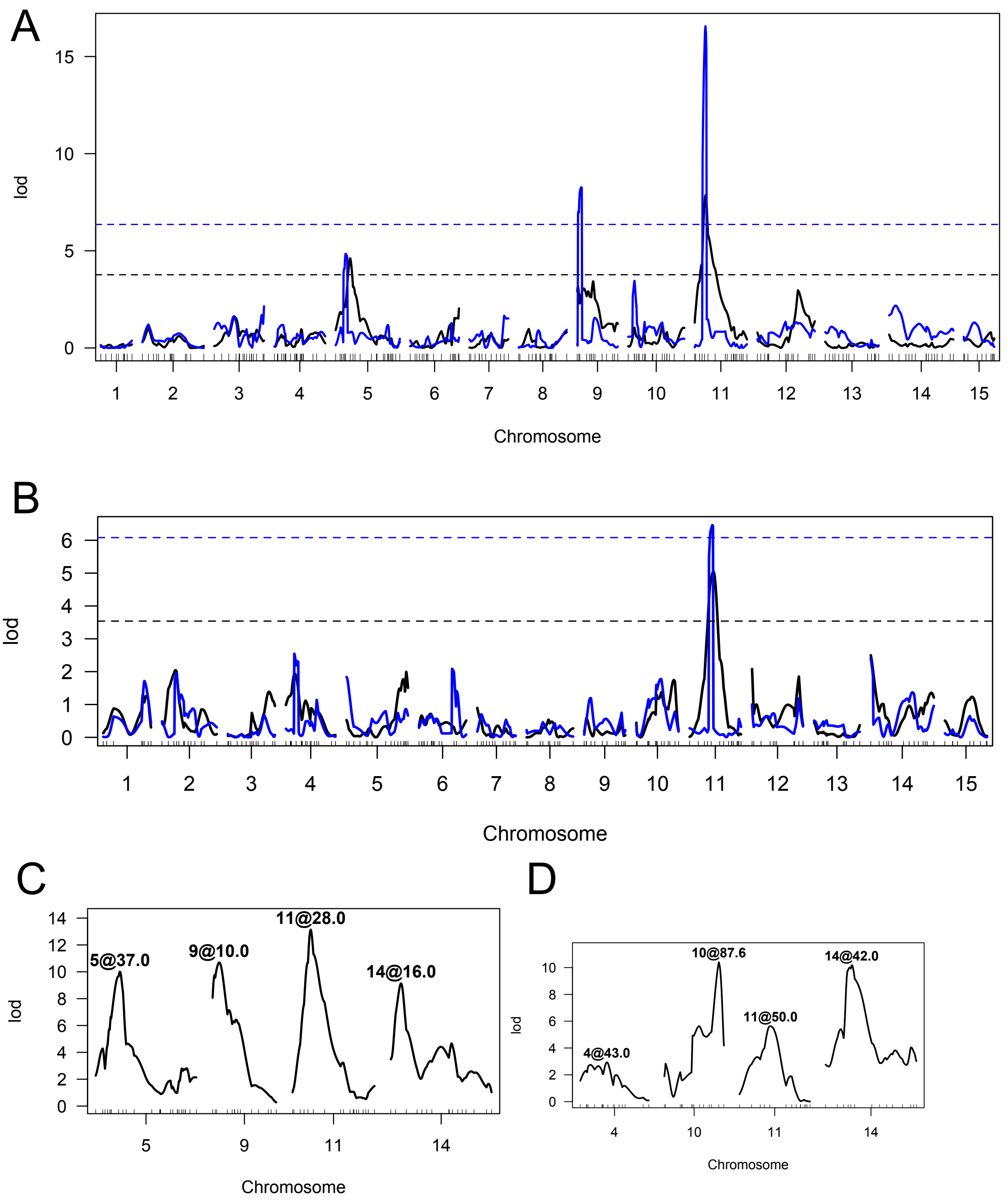

Supplemental Fig. 3. The LOD curves of QTL analyses for days from sowing to flowering in F_2 populations derived from the cross, Q65 × TKS.

A–D show LOD curves of the F_2 populations sown respectively on May 20, 2011 (**A** and **C**) and on June 7, 2012 (**B** and **D**). **A** and **B**: Black lines and blue lines respectively show the results of interval mapping (IM) and composite interval mapping (CIM). Black and blue horizontal dashed lines respectively show the permutation-test significance threshold (n = 1000, P = 0.05) of IM and CIM. **C** and **D**: LOD profiles of QTLs of the multiple QTL models. The numbers in front of "@" indicate chromosome numbers and the numbers after "@" indicate the position (cM) of QTLs.

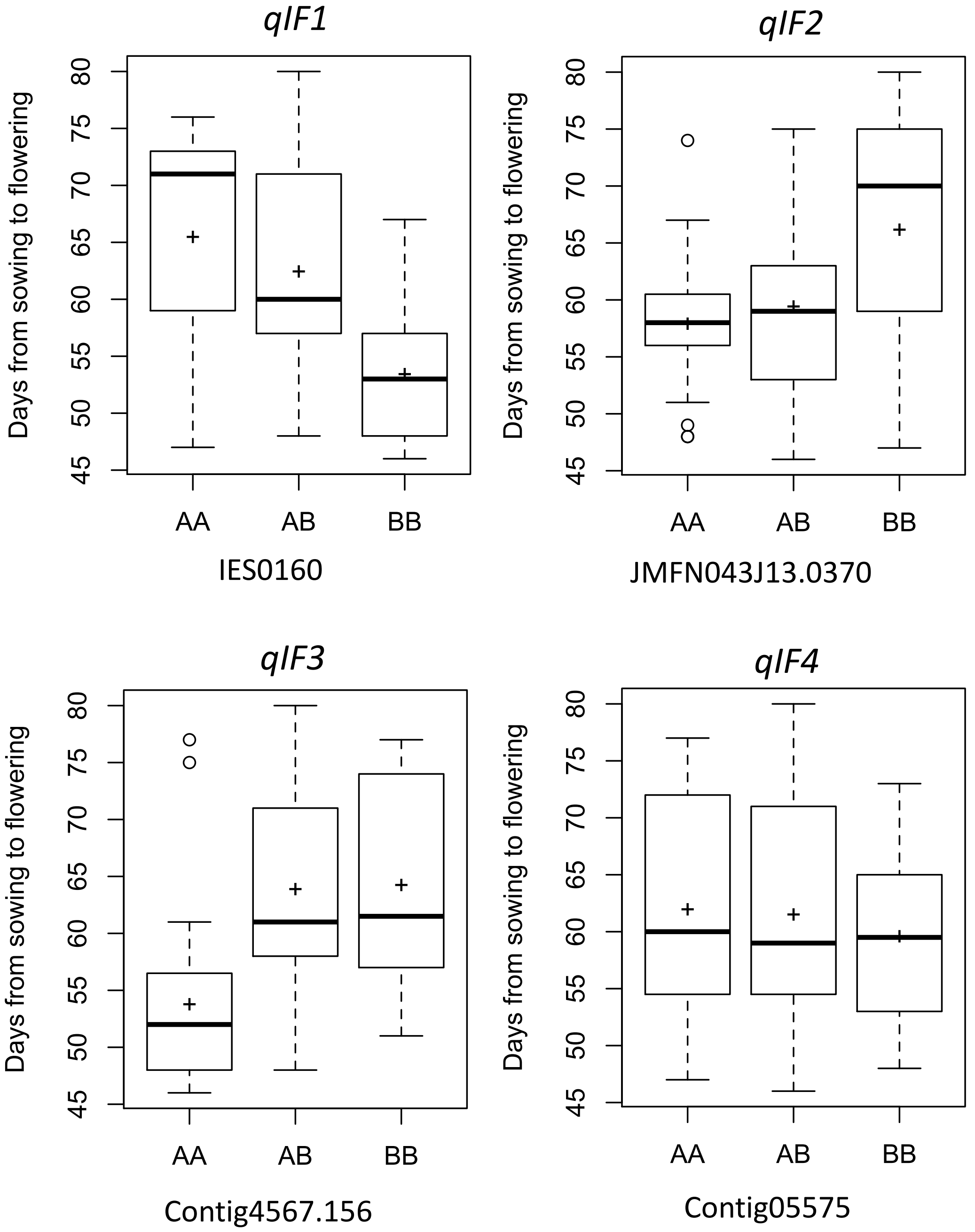

Supplemental Fig. 4. Effects of QTLs genotypes for days from sowing to flowering in the 2011 population. AA, AB, and BB of the horizontal axis respectively show TKS allele homozygotes, heterozygotes, and Q65 allele homozygotes. The names of DNA markers are shown under the genotypes. The second-nearest marker of *qIF2*, JMFN043J13.0370, was used for the box plot because the nearest marker, Contig11987, is a dominant marker and cannot distinguish between TKS allele homozygous and heterozygous effects.

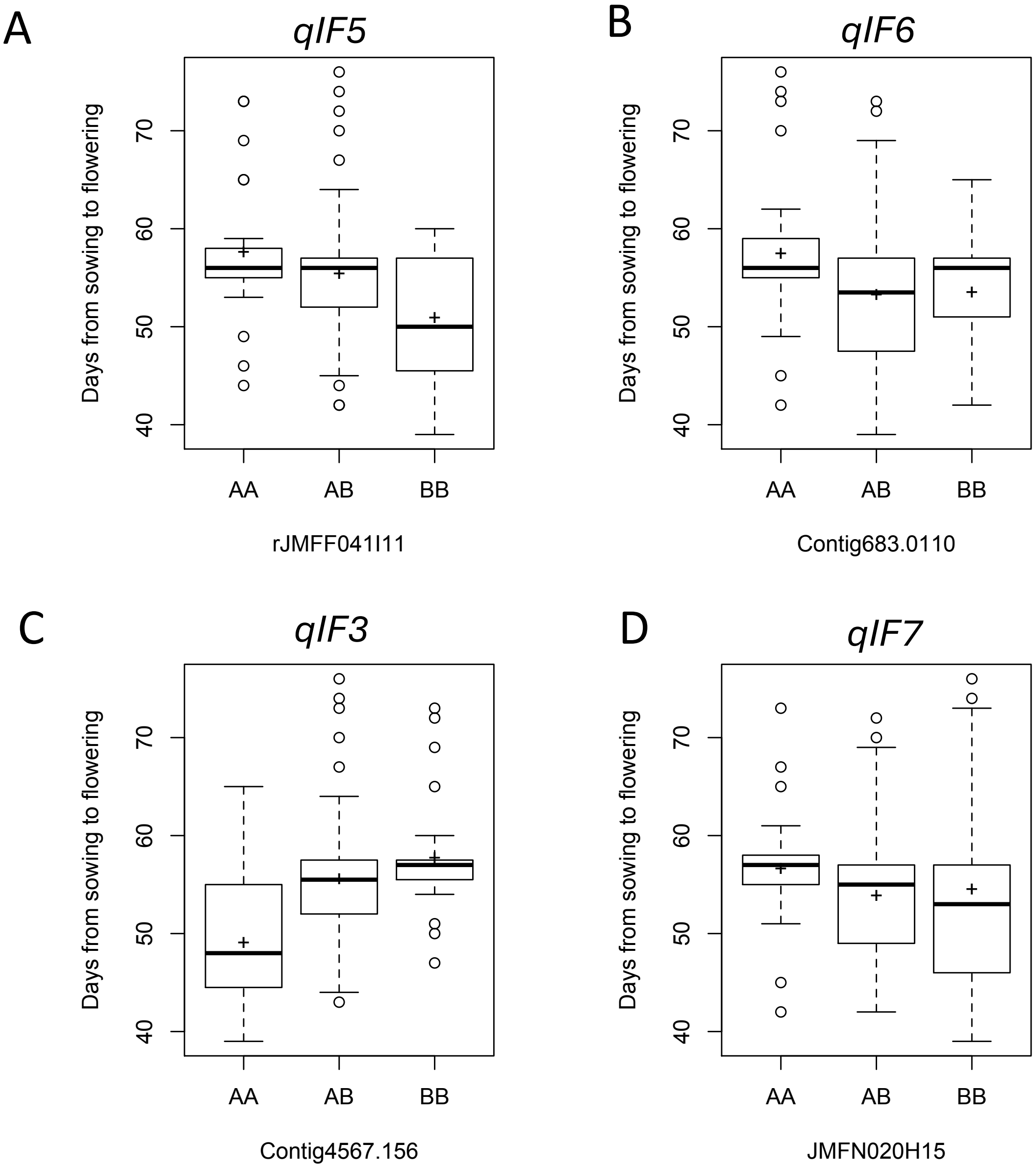

Supplemental Fig. 5. Effects of QTLs genotypes for days from sowing to flowering in the 2012 population. AA, AB, and BB of the horizontal axis respectively show TKS allele homozygotes, heterozygotes, and Q65 allele homozygotes. The nearest DNA markers for each QTL are shown below the genotypes.

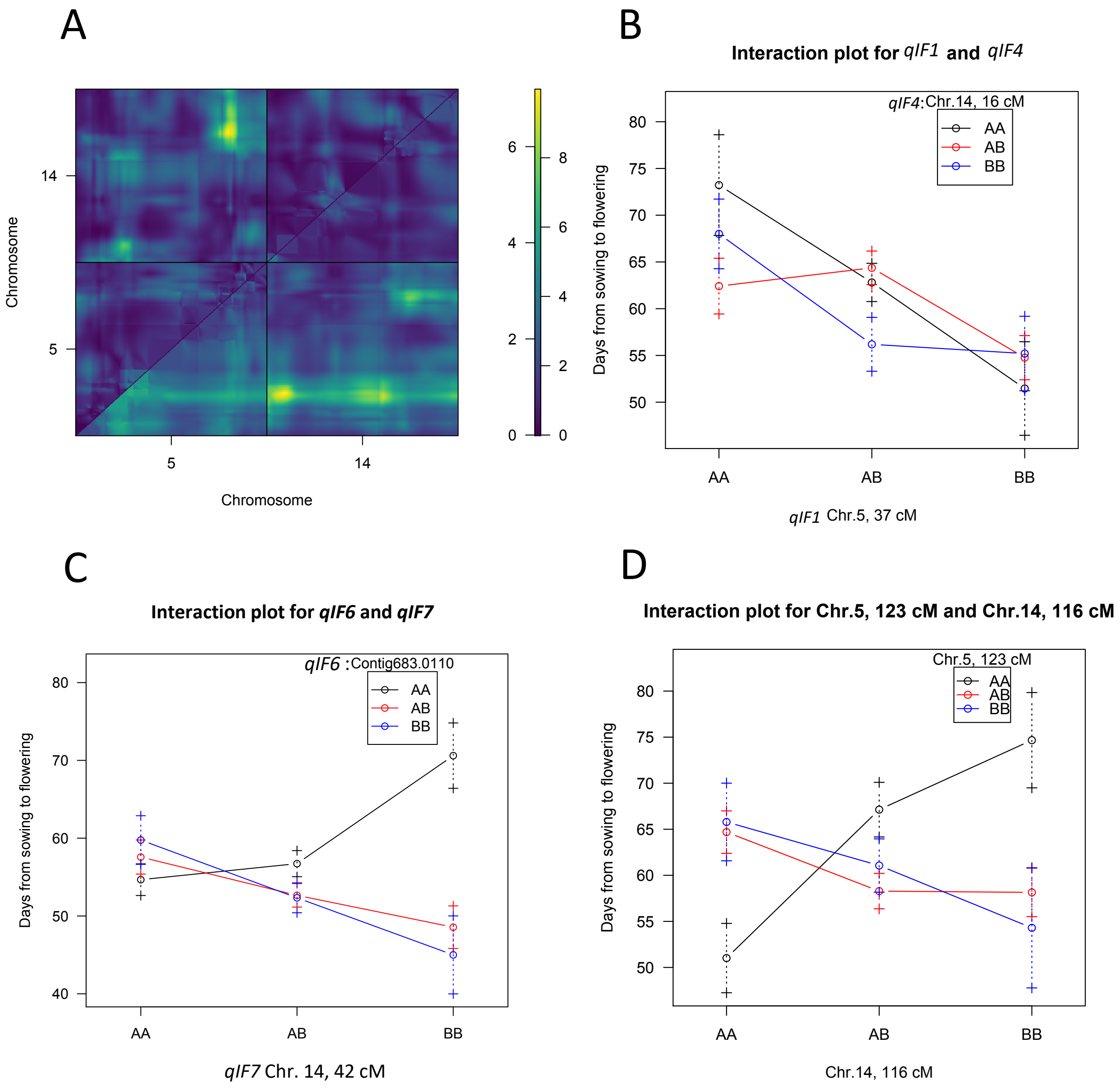

Supplemental Fig. 6. Interaction between QTLs. (A) A plot of a two-dimensional, two-QTL genome scan between chr. 5 and chr.14 in the 2011 population. The upper-left triangle contains the epistasis LOD scores ("int"), and the lower-right triangle contains the LOD scores for the full model ("full") in R/qtl. The significant threshold (P = 0.05) of LOD scores calculated through a permutation test (n = 1000) for the full model and for the epistasis are 9.31 and 6.47, respectively. (B, C and D) Plots of days from sowing to flowering against genotypes at two pairs of loci. Plots were derived from QTL analysis in the 2011 population (B and D) and the 2012 population (C). (B) Plots of *qIF1* and *qIF4*. (C) Plots of *qIF6* and *qIF7*. (D) Plots of two loci at 123 cM on chr. 5 and 116 cM on chr. 14.

```
TKS     ACGGTTGTAGCAGTGCAGAACGAAAGAAAGAAGACTAAGTGCGTGGCAATGCACATCCAA 60
Q65     ACGGTTGTAGCAGTGCAGAACGAAAGAAAGAAGACTAAGTGCGTGGCAATGCACATCCAA 60
        ************************************************************

TKS     ACACGTATACTTTCTGCCTTGGATAAACATGCTTTACCAATGAAAATTATTAGCAATAAG 120
Q65     ACACGTATACTTTCTGCCTTGGATAAACATGCTTTACCAATGAAAATTATTAGTAATAAG 120
        ***************************************************** ******

TKS     CATTTCTACATCAGATACATTTTTTGACATTGAAAGCTCACACAAACCTTTTGGAGGCCA 180
Q65     CATTTCTACATCAGATACATTTTTTGACATTGAAAGCTCACACAAACCTTTTGGAGGCCA 180
        ************************************************************

TKS     TTTACATAATTAAAAAGCTTTTGAGCTATAGAAAAAGACGTTTCAATCTGCATTTAAGGG 240
Q65     TTTACATAATTAAAAAGCTTTTGAGCTATAGAAAAAGACATTTCAATATGCGTTTAAGGG 240
        *************************************** ******* *** ********

TKS     GTTTAAATTGATTTTTTTTTTTTTTGATAAATTTTT------------------------ 276
Q65     GTTTAAATTGATTTTTTTTTTTTT-GATAAACTTTTCATTCCTTTTTTTTTTTTGAAAAC 299
        ************************ ****** ****

TKS     ------------------------------------------------------------ 276
Q65     ATTTTTTTATATTCATTTTTTTTTTTTGAATGCTACTAGCTCTATTACAATGTAGTATCT 359

TKS     ------------------------------------------------------------ 276
Q65     GTTCGTATACTTTCTCAACCTACTGAAACACAAAGAGCTAATAACGCCTCCATTGAGGCT 419

TKS     ------------------------------------------------------------ 276
Q65     CGAACTCACAACCTTTTAAATGGAAATCCAACCCGGGTACCATTGGACCACATAGTCCTG 479

TKS     -----------TGTTCCTTATTCTTACTCCTATATACTTTTACATATATTTATTTAAGAA 325
Q65     GTATTTTTTTATATTCCTTATTCTTACTCTTATATACTTTTACACATATTTATTTAAGAA 539
                   * **************** ************** ***************

TKS     AAAAGTTCCAATAGACACCTCAACTCATCAATTTGATGTAATTGAGTTGATTAGCCAATT 437
Q65     AAAAGGTCAAATAAA-ACATCAAATTATTA-TTTGATGCAATTGAGTTGATTAGCCAATT 597
        ***** ** **** * ** **** * ** * ******* *********************

TKS     AACTTTAGATAATCAATACAAAGTGACATTTTAAATTTTTAATTCAAATAAATTAATTTA 497
Q65     AACTTTAAATAATTAGTATAAAGTGACATTTTAA--------TTCAAATAAAGTAATTTA 649
        ******* ***** * ** ***************        ********** *******

TKS     AAAATTTAAAAATGTTTCTTAGAGATGCTAGCCACAGCTTCCCCGTTGGACGAAGAAGTC 557
Q65     AAAATTAAAAAATATTTCTTAGAGAAGCTAGTCATGGCTTCCCCGTTGGACGAAGAAGCC 709
        ****** ****** *********** ***** **  ********************** *

TKS     CTTCTTCATTCAAATCTGGATAGAGAAGCAGACAATAATATATTTTCTTTTCAAAAAAAA 617
Q65     CTTCTTCATCCAAATCTGAATAGAGAAGTAGACAAAAATATATTTTCTT---AAAAAAAA 766
        ********* ******** ********* ****** *************   ********

TKS     AATCATTTGAAAAAGCTTACACCGTCAATGCATTTTATTGATTTCTACCTTGAGTTTACC 677
Q65     AATCATTTGAAAAAACTTACCACGT-----CATTTTATTGATTTCTACCTTTAGTTTATC 821
        ************** *****  ***     ********************* ****** *

TKS     AGCCAATTGACTTAATAACCCTTAATTACATCAATTTGATCAACTATTAGTTTAATTGTA 737
Q65     GGCCATTTGATTTAATTACA-TCAATTTGAGTCATTTGACCAACTATTACCTTAATTGTA 880
         **** **** ***** **  * ****  *   ****** *********  *********

TKS     TGAATTTGACGAGTTAAAGTGTTCATTTTTATAATTTAAGAAGCTAAGTTATTTGGCCTA 797
Q65     TCAATTTGACGAGTTAAAGTGTTCATTTTTATAATTTAAGATGCTAAGTTATTTTGCCTA 940
        * *************************************** ************ *****

TKS     TAATTTGAGAGCTTAGCTATATGATCTTCTCCTTTTTATTTATATTTATAGTGTATATAT 857
Q65     TAATTTGAGAGCTTAGCTATTTGATCTTCTCCTTTTTATTTATATTTATGGAGTATATAT 1000
        ******************** **************************** * ********
```

```
TKS    ACCCGAATATTACTTTTAATTATAAAACTAGTAAACAGTTTATATTTATTCAGAAAAGTT 917
Q65    ACCTGAATATTACTTTTAATTATAAAACTAGTAAACAATTTATATTTATTCAGAAAAATA 1060
       *** ********************************* ******************* *

TKS    ACAAACATACCCTATATTTTTTATATTTTATGTTTT--TTTTTAT-TATTTTTATTTCCA 974
Q65    ACAAACGTACTCTATATTTGTTATATTTTGTGTTTTGATTCCCATATATATATATATATA 1120
       ****** *** ******** ********* ******  **   ** *** * *** *  *

TKS    ------------------------------------------------------------ 974
Q65    TATATATATATATATATATATATATATATATATATATATATATATATATANNNNNNNNNN 1180

TKS    -------------------------------------AATATATATATATATATATATAT 997
Q65    TATATATATATATATATATATATATATATATATATATAATATATATATATATATATATAT 1240
                                            ***********************

TKS    ATATATATATATATATATATATATATATATATATATATATATATATATATAT-------- 1049
Q65    ATATATATATATATATATATATATATATATATATATATATATATATATATATATATATAT 1300
       ****************************************************

TKS    -----------------------GTTATCTATTTTC--------CAGATCGCATCAAGGA 1078
Q65    ATATATATATATATATAATTATTGTTATCTATTTCCATGCTACCTAGACCACATCAAGGA 1360
                              *********** *         *** * *********

TKS    AAGCTCATAAGAACAGGTTCATACCAGCGCTCACACGTCGTCTATTAATTATGAAAAAGG 1138
Q65    AAACTCATAAGAACAGGTTCATACTAGGACTCACACTTCGTCTATTAATTAGAAAAAGTG 1420
       ** ********************* **  ******* **************  ****  *

TKS    CCAAATAAATCATTAAATTTTTACACTTTGTACAATAGAACCATCAAATAAAAAAGTG-- 1196
Q65    TAAAATAGACCACTGAACTTTTACAATTTTTATAATTGAATTATAAAATTAAAAAGTGCA 1480
         ***** * ** * ** ******* *** ** *** ***  ** **** ********

TKS    ---AGGCCATTAAAAAAATAAAAATTGTGCAAATAACATTCTTTACAAATTATTTCTAAG 1253
Q65    ATTGGAGCATCAAAAAAATAAAAATTGTGCTAATAACATTATTTACAAATTACTTCTATG 1540
           *  *** ******************* ********* *********** ***** *

TKS    TTTCTAGTAAAATTTGATGTCATAATGTTAAATTAAATATAAAAATAATT---------- 1303
Q65    TTTCTGGTAAAAGTTGATGTTATAATGTTAAATTATATATAAAAATTATTTTAATTATTA 1600
        ***** ****** ******* ************** ********** ***

TKS    --------------------AAAAAAAAATCAAAAGAAAGTTCATCTACTGTGGATAAAG 1343
Q65    TTTAAAAATATTTTTTAAAAAAAATAAATTCAGAAAAAATTTCATCTGTTGTGAATGAAA 1660
                           **** *** *** ** *** *******  **** ** **

TKS    GGAAAGGCCGTCATGGCTGCTCCCACCTTCGTC---------------------CAA--- 1379
Q65    GGAAAAGTAGCTATGACTGCCCCTACCTTCGTCTGCTATGGATGGAGGGGGAGGCAACCA 1720
       ***** *  *  *** **** ** *********                     ***

TKS    -------------TTTGAACGAAGGGGAGGCAACCATGGCTGCTCCCTCCTTCATCCAAA 1426
Q65    TGGTTGCCCCACCTTTGGACGAAGGGGAGGCAGCCATGGCTGCTCCCTCCTTCATCCAGA 1780
                    **** ************** ************************* *

TKS    TCGGATAAAGGAGGGGGCAACCATGGCTGCCTCCATCCTCGTCCAAAACAAAGGTGAGGG 1486
Q65    TCGGACGAAGAAGGGGGCAACCATAGCTGCCTCCATCCTCGTCTAGGACAAAGGTGGGGA 1840
       *****  *** ************* ****************** *  ********* **

TKS    CAACCATGGTTATCATTCCCTTCATCCACAACAAACGAAGGTGTAGGCAATCATGCCCTT 1546
Q65    CAGTAATGGTTGTC--TCCCT------------GATGAAAGGGTAGGTAGTCGTGCCCTT 1886
       **   ****** **  *****             * *** * ***** * ** *******
```

```
TKS     ACACCTT---TTAAAATTAAATTTTTT-------------TTTAAAATAATAATTAAAAT 1590
Q65     CCACCTTCTTTTGAAATTAAATTTTTTGTTTTTAATTTTTTTTAAAATAATAATAATAAT 1946
         ******   ** **************             ************** * ***

TKS     AAT-----------TTATATATATACACCATA---ATAATTTAACATTATGACATTATAT 1636
Q65     AATAATAATAATAATAATTAAAATAATTTATATATATAATTTAACATTATGACATTATAT 2006
        ***           * **  * ***    ***   *************************

TKS     TTAATAGAAACTTGAAAATAACTTATAATAAAATACTATTTGTATAATTTTTGAGTTCTT 1696
Q65     TTAATAGAAACTTGAAAATAACTTATAATAAAATACTATTTGTATAATTTTTGAATTCTT 2066
        ****************************************************** *****

TKS     AATAATTTAATTACATATTTTTTTTGTTCAATGATCAATTACACACAATGTAAAAGTTCA 1756
Q65     AATAATTTAATTACATATTTTTTTTGTTCAATGATCAATTACACACAATGTAAAAGTTCA 2126
        ************************************************************

TKS     ATGGCCTATATGACATTTTTTTCACTAATTTGATGTGAAGAACGTTTCTCCCAGAGTGTG 1816
Q65     ATGGCCTATATAACATTTTTTTCACTAATTTGATGTGAAGAACGTTTCTCCCAGAGTGTG 2186
        *********** ************************************************

TKS     GGCAGCAAACGGATTCCAAAAGCCAAAGCCATTGACAGGACAGGTCTGTAAAACAGAATC 1876
Q65     GGCAGCAAACGGATTCCAAAAGCCAAAGCCATTGACAGGACAGGTCTGTAAAACAGAATC 2246
        ************************************************************

TKS     TGGGTCAGCGGGACCCCGCGGCTGGCCCCACCAGAATCACAGCCAGCCACAAGATAATAC 1936
Q65     TGGGTCGGCGGGACCCCGCGGCTGGCCCCACCAGAATCACAGCCAGCCACAAGATAATAC 2306
        ****** *****************************************************

TKS     AACCTTTTGCAACCCGCTAATATCGCACCACGTGTCAGCTCTGGATTCTCCTCCCACTCT 1996
Q65     AACCTTTTGCAACCCGCTAATATCGCAACACGTGTCAGCTCTGGATTCTCCTCCCACTCT 2366
        *************************** ********************************

TKS     CCCTTCCAACTTGCTCACTGCTACCACAAAGTCATAAAAAGCATAGCTGCAGGACTCGGT 2056
Q65     CCCTTCCAACTTGCTCACTGCTACCACAAAGTCATAAAAAGCATAGCTGCAGGACTCGAT 2426
        ********************************************************** *

TKS     CACTCTAAATAAATACGTTTGAGTGTGTGTAGTCTTACGTGTGTGCGAGGAAACACTCAA 2116
Q65     CACTCTAAATAAATACGTTTGAGTGTGTGTAGTCTTACGTG----GGAGGAAACACT-AA 2481
        *****************************************     *********** **
```

Supplemental Fig. 7. DNA sequence alignment of the 5′ flanking region of *InCO* between TKS and Q65. Yellow highlights denote a SINE-like sequence. Light blue highlights denote target site duplications. A bent arrow indicates a putative transcript-start site.

```
TKS_ni    MLKEESCEVLDLDVTIGSSSGSRSGNKQNWARVCDICRSAACSVYCRADLAYLCGGCDAR 60
Q65_si    MLKEESCEVLDLDVTIGSSSGSRSGNKQNWARVCDICRSAACSVYCRADLAYLCGGCDAR 60
          ************************************************************
                           B-Box zinc finger
TKS_ni    VHGANTVAGRHERVLVCEACESAPATVICKADAASLCAACDSDIHSANPLARRHHRVPIL 120
Q65_si    VHGANTVAGRHERVLVCEACESAPATVICKADAASLCAACDSDIHSANPLARRHHRVPIL 120
          ************************************************************

TKS_ni    PISGTLYGPPTSNPCRESSMMVGLTGDAAEEDNGFLTQDAEETTMDEDEDEAASWLLLNP 180
Q65_si    PISGTLYGPPTSNPCRESSMMVGLTGDAAEEDNGFLTQDAEETTMDEDEDEAASWLLLNP 180
          ************************************************************

TKS_ni    NPNPNPNP----VKSNNSTNMCKGGNNNNN---EMSCAVEAVDAYLDLAEFSSCHNNLFE 233
Q65_si    NPNPNPNPNPNPVKSNNSTNMCKGANNNNNNNNEMSCAVEAVDAYLDLAEFSSCHDNLFE 240
          ********    ************.*****   **********************:****

TKS_ni    DKYSINQQQNYSVPQRNMSYRGDSIVPNHGKNQFHYTQGLQQHNHHAIFNCKEWNMRILT 293
Q65_si    DKYSINQQQNYSVPQRNMSYRGDSIVPNHGKNQFHYTQGLQQHNHHRNFQLQGMEYENFN 300
          **********************************************  *: :  : . :.

TKS_ni    R---------DMVSISSMDVGVVPESTLSDTSISHSRASKGTIDLFSGPPIQMPPQLQLS 344
Q65_si    TGYGYPASISHTVSISSMDVGVVPESTLSDASISHSRASKGTIDLFSGPPIQMPPQLQLS 360
                    . ******************:*****************************
                              CCT domain
TKS_ni    QMDREARVLRYREKKKTRKFEKTIRYASRKAYAETRPRIKGRFAKRTDVDTEVDQIFYAP 404
Q65_si    QMDREARVLRYREKKKTRKFEKTIRYASRKAYAETRPRIKGRFAKRTDVDTEVDQIFYAP 420
          ************************************************************

TKS_ni    LMAESGYGIVPSF 417
Q65_si    LMAESGYGIVPSF 433
          *************
```

Supplemental Fig. 8. An amino acid sequence alignment of the InCO/IhCO protein, encoded by the transcript variants, *InCO* (ni) of the TKS allele and *IhCO* (si) of the Q65 allele. Sequences were aligned with Clustal W (Ver. 1.83, 2003). Blue highlighted boxes show two tandem B-Box-type zinc finger motifs. Yellow highlighted boxes show CCT domain.
