# Supplementary material for: Detection and validation of QTLs for flowering time in morning glory
Source: Breed Sci. 2025 Oct 15;75(5):339–48. doi: 10.1270/jsbbs.24067 (PMC13129579; doi:10.1270/jsbbs.24067)
Supplement: Supplementary file 2 — Supplemental Tables [file 75_339_s2.pdf]

doi: 10.1270/jsbbs.24067

Supplemental Table 1. List of EST-SSR markers derived from *I. batatas*

Marker name	Core motifs	Chromosome	Forward primer (5´ to 3´)	Reverse primer (5´ to 3´)
IES1076	AAC	2	TAGAGCCTTTGACGTCCGAT	TAATTTTCTCCAATTGCCGC
IES0411	ATC	3	AGGAAGCCCAATGGAGTTTT	AGTCTGGGTTGGCAATTACG
IES0856	GGA	4	TTGCTGATTCGGACACTGAG	CACTCCCAAGAAGTTGCTCC
IES0575	AG	4	GCTGATAACACCTGTGAGAGATAGA	TGTGTTCAATTTCACCGGAA
IES0160	ATC	5	AACGGCCAGAGATTGTGAAG	CATGGCAAATCATCGTCATC
IES0847	AAG	5	CGGATCACTGAGATGAGGGT	TGTGAGCCATTCTGCGTAAC
IES0809	ATC	8	AAGCACCTACTCCTGCTCCA	TCCACCCTGCTCTAGGTACG
IES0902	AAG	9	CGTAGCTCTCCCCTCTCCTT	TGCTTGTTGCTGTTTCAACC
IES0410	AAG	10	CAGGGGCATAACCGTAATTG	ATGATCCCCAGGTTTAAGGC
IES0433	ATC	12	CCCATTTCTCCACAGGGATA	GAAGGGCAGGAACAAATTCA
IES0874	ATC	12	AGATGCACTCCCTTGCTTTC	ATGGGGAGGAGATCCAAGTT
IES0414	AG	12	TGCATTTCTCTCTGTTCCCC	GTAAAAATCCCCGTGCTGAA
IES0315	GGC	13	CTATAGGTTGGCACCGGAAA	TTGTGGGCTATATTGGCTCC
IES0680	AAG	13	GGGTTTTCAGAGACCGTTGA	CCATGCACACCTTCACAAAC
IES0536	AAG	13	ATGTTGCCTAATGGACAGGC	AATGGGACAAATGGCAACTC
IES0862	ATC	14	TCGTTGGGACTCCTGATACC	AGTTGTCGCTTGCGTTTCTT
IES0177	GGA	15	CAAACATGGACGAAATCACG	TCACCGTCGTCATCGTACTC

Supplemental Table 2. HRM-SNP and SSR markers developed and used for genetic linkage-map construction and QTL analysis of DTF in *I. nil* and *I. hederacea*

Marker name	Marker type [a]	Chromosome	Forward primer (5′ to 3′)	Reverse primer (5′ to 3′)
rJMSF039K04.591	HRM	1	TCTTCCTGTGCAACTTACGC	AAATTGCTCCATTTGGCTTG
Contig781.719	HRM	1	AACTCTTGCTCGAGCCATTT	CACTCAGGTTCCTGATTTTCTAA
fe-SSR	SSR	2	TCAAACATCGACCTCGTGAC	GCCCTCCATCTCTCTTCACA
Contig1578.447	HRM	2	TGAAGGATTGGCGAGAAATC	GTCGGGCCACCTTGAATAAT
Contig8667.442-m	HRM	3	CGTGTCCTTGTCGTTTTCATT	CCCTTCAGCTCTTCCCTCTT
MYBX	HRM	3	GCCATCGCCAACATTTTAC	TTGATCTAGATTGTGGGCAAGA
Contig1617.421-dy	HRM	3	CCATGGACAAAACCCAGAAC	CGGACTGATGGTGCTGTAGA
Contig1620.744	HRM	3	CACGACCAAACAGCTCTTCA	CGTACAGATCGACCTGATTGC
PnFT2	HRM	3	ATCAAATAGGTTGCCGGAGA	CGACGACCAGTGGGTCTCT
Contig10056.454	HRM	4	CTCCAATCATCTTCAACACCAA	CACGACATGATCGTTCCTCA
InPHYC	HRM	4	CTGCACTTGAAAAAGCAGCA	GCTTACCCGAATTTTTGCAG
Contig2109.295-mg	HRM	4	GCCGCTCAGTTCTTGAAAGT	GCGAAGACGAGGTCTTGATAG
Contig5247.271-a3	HRM	5	AAAGCCGACACGAATTTGAC	TGGCTTCATCAAAGCTTCCT
InMYB2	HRM	5	ATGGTTACATGTGTTTAGGTGGTC	CTTCACATCGTTCGCTGTTC
Contig12385.595	HRM	5	ATCTTCATCGGTGACGCTTT	GTGGTCCCCGGAGACTTT
Contig7414.299	HRM	5	AACACCATGGGAGATTCGAG	GGCAAGATCGATTTCTTCGT
Contig6884.391	HRM	5	TGGTGGCACAATAATTGACATT	AGCCAGCACAGTGTTTTTGA
Contig126.513	HRM	5	GTCGCTTCGCAAATCCTAGA	TATCTGATGGCCAAGGGTTG
Contig11461.715	HRM	5	CGACAATCCAAAATCTGCAA	ACCTGCACACTGAAGCTCAC
Contig7935.1121	HRM	5	CTGCTGTAAGGCCTCCACCT	AAGGGAGATTCGCGCTAAAG
Contig1107.272	HRM	5	CAGATGCAGCCCAATTGATA	TCCAATTTCAATGGTCCTTTT
InG31484728	HRM	5	GGAGATTACAGTTCCGACGTC	TTTGTCCTCCTCCGTCGAC
Contig10004.219-efp	HRM	5	TTTCTCCAAATAAAGTACACTGCAA	GACACAATTTCTGGGTCCAA
DP-SSR	SSR	6	ATGGTGGCATGGACAATCTT	CCTTCACCCATGTAGTTCCTG
InPHYE	HRM	6	GGCCATAGCTCAGTACAATGC	TCTTCTTCAGTCACATTCTGTGG
5gt7	HRM	6	GCCGGAATCTTCTGTGGTT	CAGCCCGCAGTTTATCAGC
Contig10633.676-ivs	HRM	7	GCCTTGTCCATTTTCGTCAC	TCAACGAGCGGTTCATTATC
Contig12273.307-kbt	HRM	7	CAGTCCACCTCTGGGAATCT	CGGTGAATGTATGGGCTGAT
JMFN043J13.0370	HRM	9	GCGTTTGAACTCCAGATTGC	CAAAACTCATCGGCAAACAA
PnFT1	HRM	9	CCGGCAGACAGTTTATGCAC	TCAGCAAAGTTTCGAGTGTTG
PnTFL1a	SSR	10	GAGAACAAGAAGCCCCTCAA	ACTGTGGGAGTGAAGGCATC
Contig1647.128-pr	HRM	10	GAGAGAGTCACGTTAATCCTGAGA	GTGGGGGTAGTGTTGAAGGA
Contig3344.254	HRM	10	GCTCCTCACACCTGTCATTT	TCTCCCCTTCTTCATCTCCA
Contig4567.156-InCO	HRM	11	CAGAAGTGGCAACAAGCAGA	GAACGGCATATGTCGCAGA
Contig4216.592-InGI	HRM	11	GGCATGCTGTTATTCATCCA	GGCAAAAGGTGGACTATTTTT
JMFF032K01.306-c1	HRM	11	TTGCAGATTGAGATGGTTGAA	GCTTATGGAGGCGTAGAATGA
Contig2647.823-sp	HRM	11	TGATATCTGTTTTATTTGGGAGTGG	CAAAATAAAAACAAAAGTAATCCGTA
rJMFF001I14.223	HRM	12	GTGTATGGAGCTGGCTTGG	GCACGCTCTCTCAAAGATGA
Contig2300.318	HRM	12	AACAGCATCCGAGTCTGGAA	CCCGTGGCTACGAGATAGTG
Contig3548.340	HRM	12	AAGCTAAGGTGTGGGAGAAGC	GTGGCATTGGTTTAGGCATT
Contig12.373	HRM	12	GGGGATTGTGATCATTCTGG	TGCCAATCTCGTAATCCTCA
Contig23.333-r3	HRM	13	ACCTGGTGAACCACGGGATA	CTGACCGAAAAACCCCTCTC
CHS-E	HRM	14	AGCGATCCAGAAAAGGGAGT	TCCTGGGCTCATATCAAAGC
JMFS127D02.495	HRM	14	ACGGATTTGAGGACCCTTTT	AAATTGGGTTGAGCAAGGTG

[a] HRM and SSR respectively represent high-resolution melt and simple sequence repeats.

Supplemental Table 3. List of developed Tm-shift primers based on the *I. nil* EST database

Marker name	Chromosome	Fwd. / Rev.	Primer sequences (5´ to 3´)
Contig71.0517	1	Forward	AAGAAGCTTTCGAGGCGGCGTA
		Reverse 1	GCGGGCAGGGCGGCGCGGCGGAATCACTGGTCATT
		Reverse 2	GCGGGCGCGGCGGAATCACTGGTCATA
Contig73.0355	1	Forward	GAAGCAGAATGTTAAGCAATCGAGGA
		Reverse 1	GCGGGCAGGGCGGCTATTCAGAGTAACAGGAGTAGGCGCG
		Reverse 2	GCGGGCTATTCAGAGTAACAGGAGTAGGCTCA
Contig213.0351	2	Forward 1	GCGGGCAGGGCGGCCGGCGAATTTGGTGACGTATCTC
		Forward 2	GCGGGCCGGCGAATTTGGTGACGTATCTG
		Reverse	CGAGTTGCTCATCTTCGCCACAT
Contig13313.0335	2	Forward	TGTGCAATAGCTTTATTCGGAACCA
		Reverse 1	GCGGGCAGGGCGGCGGAGAGCAAGAATTTCCATGACCGG
		Reverse 2	GCGGGCGGAGAGCAAGAATTTCCATGACCGC
Contig29.0586	2	Forward	TGTTTCACGCATGGGAAGTATGAGAA
		Reverse 1	GCGGGCAGGGCGGCAACTGCACTTAAGTTGGCAGGGTCCG
		Reverse 2	GCGGGCAACTGCACTTAAGTTGGCAGGGTACA
Contig117.0507	2	Forward	CGGGTGTTCTTCAACAGCTCGAC
		Reverse 1	GCGGGCAGGGCGGCCACGAGTCGGTGCCAAGACAAG
		Reverse 2	GCGGGCCACGAGTCGGTGCCAAGACCAA
Contig39.0484	2	Forward 1	GCGGGCAGGGCGGCGCTTCATCGTTTCTTACCTCGTGG
		Forward 2	GCGGGCGCTTCATCGTTTCTTACCTCGGGA
		Reverse	GTAATGCCCACGAACAGCAGAAGC
Contig341.0578	3	Forward	GCGAGATGATTTGGATAACATTTCCCT
		Reverse 1	GCGGGCAGGGCGGCAGAAGTTCTTCTCAAAGCGAACCCAG
		Reverse 2	GCGGGCAGAAGTTCTTCTCAAAGCGAACCAAA
Contig591.0478	3	Forward	GGGAAGCATTCCTGCAAAGGTTTAGA
		Reverse 1	GCGGGCAGGGCGGCGCGAGTCTCCCACAAACATAATCGTC
		Reverse 2	GCGGGCGCGAGTCTCCCACAAACATAATCTTT
Contig11523.0689	3	Forward 1	GCGGGCAGGGCGGCCTAAACCTTCCTTGGTTGTGAGGCGG
		Forward 2	GCGGGCCTAAACCTTCCTTGGTTGTGAGGAGA
		Reverse	ACAACCGGAAGGTCAAGAAAATCTGG
Contig75.0423	4	Forward 1	GCGGGCAGGGCGGCAAAATGCTGAGAAACGTGGTGCAC
		Forward 2	GCGGGCAAAATGCTGAGAAACGTGGTGAAT
		Reverse	CAAATTCGGGCAACTGGGGATTAT
Contig132.0440	4	Forward 1	GCGGGCAGGGCGGCGCGCAGTCGTACATGGATGACTTG
		Forward 2	GCGGGCGCGCAGTCGTACATGGATGACGTA
		Reverse	AATGAAACTGCCAAGAACCAGGGC
Contig210.0518	4	Forward	CGAAGGTGGCAAAATATATGGGGTG
		Reverse 1	GCGGGCAGGGCGGCCAATTGCAAAAGGCTGCTCCAAGAC
		Reverse 2	GCGGGCCAATTGCAAAAGGCTGCTCCAATAT
Contig10534.0459	4	Forward	GATACTGAGGTCGGTGGGAATCAAGG
		Reverse 1	GCGGGCAGGGCGGCTGCTGATACTAAGGTCCCTGGTATGG
		Reverse 2	GCGGGCTGCTGATACTAAGGTCCCTGGTAGGA
Contig618.0053	4	Forward	CGCCCCTCTCCTCCTCCTCTTA
		Reverse 1	GCGGGCAGGGCGGCCATGGAAACGGCGAACTGG
		Reverse 2	GCGGGCCATGGAAACGGCGAACGGA
Contig312.0049	5	Forward 1	GCGGGCAGGGCGGCACATTCTCCGCCGTTTTCTGAGTGTC
		Forward 2	GCGGGCACATTCTCCGCCGTTTTCTGAGTTTT
		Reverse	ATTGGGGATTGGATTGTGATGTATGC
Contig9549.0185	5	Forward 1	GCGGGCAGGGCGGCCATTCATGGCAGCTTATGCAACAGC
		Forward 2	GCGGGCCATTCATGGCAGCTTATGCAACAGG
		Reverse	CGAGAACCTTCTCCAGAGCCACTTC
Contig11034.0239	5	Forward	TGCATATGCTACAGGAATTTCTCCCA
		Reverse 1	GCGGGCAGGGCGGCCGTTTGATGGTCATACCATGCATTCC
		Reverse 2	GCGGGCCGTTTGATGGTCATACCATGCATGCT
Contig10043.1070	5	Forward	GTCGGTAATGAGGTTCTGGCGAAGAG
		Reverse 1	GCGGGCAGGGCGGCTTTCCCACCTCTCCAATCTCAACCAG
		Reverse 2	GCGGGCTTTCCCACCTCTCCAATCTCAACAAA

Supplemental Table 3. (continued)

Marker name	Chromosome	Fwd. / Rev.	Primer sequences (5´ to 3´)
Contig223.0210	5	Forward 1	GCGGGCAGGGCGGCATGCGAGTTGTTTGAATTGGACTGG
		Forward 2	GCGGGCATGCGAGTTGTTTGAATTGGACGGA
		Reverse	TGATCGAAAATCAATAGCACACAAA
Contig1732.1500	6	Forward	GACCTGCCGGAGAAGGTTTTGTTAGA
		Reverse 1	GCGGGCAGGGCGGCACACATTCTCTCTGTGTCCAAAGTCG
		Reverse 2	GCGGGCACACATTCTCTCTGTGTCCAAAGGCA
Contig348.0441	6	Forward	GCCGCAGCCTCGAATTGTTAGT
		Reverse 1	GCGGGCAGGGCGGCGGCGCAATGAGTGGTAAAATCG
		Reverse 2	GCGGGCGGCGCAATGAGTGGTAAAAGCA
Contig638.0218	6	Forward	CGATGTGCCGGTGATTGACCTAC
		Reverse 1	GCGGGCAGGGCGGCCCGAATCTCCCCACTCCTCAGAG
		Reverse 2	GCGGGCCCGAATCTCCCCACTCCTCAGAC
Contig10207.0412	6	Forward 1	GCGGGCAGGGCGGCCCATGTGTTCTCGCTCCCAAATAGG
		Forward 2	GCGGGCCCATGTGTTCTCGCTCCCAAATAGC
		Reverse	AAGCAGAAAATCCCATGTCCAATCG
Contig13203.0679	7	Forward 1	GCGGGCAGGGCGGCGCTGTTGACGCTTCAAAATGCTTGTC
		Forward 2	GCGGGCGCTGTTGACGCTTCAAAATGCTTTTT
		Reverse	ACTGATGAGAGAAGCACGGACAGCTT
Contig7688.0677	7	Forward 1	GCGGGCAGGGCGGCGCACTGCCTAGCTCAGCCTCCTCTA
		Forward 2	GCGGGCGCACTGCCTAGCTCAGCCTCCTCTT
		Reverse	TTGTAGGTGCCAATACCAATGCCAA
Contig361.0384	7	Forward	TTCGCTACTTCATCCACCAGTTTTGG
		Reverse 1	GCGGGCAGGGCGGCCAGCAGAAGAACCAAAAGAAAATTCG
		Reverse 2	GCGGGCCAGCAGAAGAACCAAAAGAAAATGCA
Contig13068.0510	7	Forward 1	GCGGGCAAGTTGCTCCAAGAAGAACAGGGAGA
		Forward 2	GCGGGCAGGGCGGCAAGTTGCTCCAAGAAGAACAGGGCGG
		Reverse	CTCCACAAAGCATTCCCTTCACAGAC
Contig45.0110	8	Forward 1	GCGGGCAGGGCGGCAAACCGGCAAAGAAAGGTGGGGAC
		Forward 2	GCGGGCAAACCGGCAAAGAAAGGTGGGGAG
		Reverse	TTTGACGGGAAATCATCGGAAAAA
Contig12633.0348	8	Forward 1	GCGGGCAGGGCGGCCTTGATATGGCTTGCCGATGAACGTC
		Forward 2	GCGGGCCTTGATATGGCTTGCCGATGAACTTT
		Reverse	GCAGAAGGATCTGTTGGAGACAATGG
Contig14002.0191	8	Forward	TCAAACCCTCCAGAACCTCCCTTTT
		Reverse 1	GCGGGCAGGGCGGCTGGTGATGAAGAAGATTGCTCGTCG
		Reverse 2	GCGGGCTGGTGATGAAGAAGATTGCTCGTCC
Contig6146.0392	9	Forward 1	GCGGGCAGGGCGGCCACCCTCGATAATTCCTTCCAACTCA
		Forward 2	GCGGGCCACCCTCGATAATTCCTTCCAACTCT
		Reverse	AAAATACCCGATTCCGCATGAAATTG
Contig502.0119	9	Forward	CCAGTGATTGATTGATCTCTCGTTACCA
		Reverse 1	GCGGGCAGGGCGGCTCTGCATCTTGCCTCTCTTTCTCGTC
		Reverse 2	GCGGGCTCTGCATCTTGCCTCTCTTTCTCTTT
Contig572.0192	9	Forward	AGGGGGAGAAGATGAAGGAGAGAACC
		Reverse 1	GCGGGCAGGGCGGCACTTCCACTGTGTGTAGCGCTCGGTC
		Reverse 2	GCGGGCACTTCCACTGTGTGTAGCGCTCGTTA
Contig1098.0735	9	Forward	TCCGTTCACGCATCCTTCCACT
		Reverse 1	GCGGGCAGGGCGGCTGACCCGGTTCTTCAGATCAGG
		Reverse 2	GCGGGCTGACCCGGTTCTTCAGATCCGA
Contig31.0114	10	Forward	AGAAACCAAAACGGAACAAATACGCC
		Reverse 1	GCGGGCAGGGCGGCAAGTCATGGAAGCTAAGAGAGAGAAG
		Reverse 2	GCGGGCAAGTCATGGAAGCTAAGAGAGAGCAT
Contig9165.0662	10	Forward 1	GCGGGCAGGGCGGCTGCATTCTTTGGGAAAACTTGGTCC
		Forward 2	GCGGGCTGCATTCTTTGGGAAAACTTGGTCG
		Reverse	TAGTGGTTGGAACTCACGCTGTGCT
Contig52.0536	10	Forward	AAAAGTGGGCTCTTTTGTTGACAGGC
		Reverse 1	GCGGGCAGGGCGGCCAAAAAACACATGCAATCCCATTGCG
		Reverse 2	GCGGGCCAAAAAACACATGCAATCCCATTTCA

Supplemental Table 3. (continued)

Marker name	Chromosome	Fwd. / Rev.	Primer sequences (5´ to 3´)
Contig683.0110	10	Forward	GCCACAGTGACACTCCAATGTTGA
		Reverse 1	GCGGGCAGGGCGGCCCGGCCAAAGGTCCGGTAAAGTA
		Reverse 2	GCGGGCCCGGCCAAAGGTCCGGTAAAGTT
Contig514.0130	10	Forward 2	GCGGGCACGCCCCACAGTCAACGACA
		Forward 1	GCGGGCAGGGCGGCACGCCCCACAGTCAACGCCG
		Reverse	GGCAACTCGGTGAGTGAGGCTT
Contig13171.0174	11	Forward	AATCTGGGGAGTTGACCCAAAAAGC
		Reverse 1	GCGGGCAGGGCGGCCTTGGTCTCCTTGATCTACGGCTCG
		Reverse 2	GCGGGCCTTGGTCTCCTTGATCTACGGCTCC
Contig394.0210	11	Forward	TCTGGCCAAGGATAAAGCGTTT
		Reverse 1	GCGGGCAGGGCGGCATATCGGCGGCGAGGTGTGTC
		Reverse 2	GCGGGCATATCGGCGGCGAGGTGTGTG
Contig10076.0370	11	Forward	CCACATTCTCTAGCTGCTCGATTGCT
		Reverse 1	GCGGGCAGGGCGGCAGTACTTCGACATGCCACCTTTGCCG
		Reverse 2	GCGGGCAGTACTTCGACATGCCACCTTTGACT
Contig529.0215	11	Forward 1	GCGGGCAGGGCGGCGTTCCGAAGCGGTGGACAGTTGAG
		Forward 2	GCGGGCGTTCCGAAGCGGTGGACAGTTTAA
		Reverse	TCTTCTTCGGCGCACTCATTTCTC
Contig43.0622	11	Forward	GCAACAGTCCATGAAGAAAGAGCGAA
		Reverse 1	GCGGGCAGGGCGGCTCTTAGTATCCACGGCGGTGGTACTG
		Reverse 2	GCGGGCTCTTAGTATCCACGGCGGTGGTAATA
Contig639.0355	12	Forward 1	GCGGGCAGGGCGGCGGGACCTAATGGTGCCGAATGCC
		Forward 2	GCGGGCGGGACCTAATGGTGCCGAATTCA
		Reverse	ATCTCACCGGCTTGGCGTACAAC
Contig50.0300	12	Forward	TTCTCTGCGCAATCAAAGCCGTA
		Reverse 1	GCGGGCAGGGCGGCTGGATTGGTTGGTCCAAGATTCC
		Reverse 2	GCGGGCTGGATTGGTTGGTCCAAGATGCT
Contig268.0442	12	Forward 1	GCGGGCAGGGCGGCCGGATATGATTGATGGATTCCAGCAG
		Forward 2	GCGGGCCGGATATGATTGATGGATTCCAGAAA
		Reverse	GTAGATGATCGGAATACCGAGCCGGG
Contig8337.0661	12	Forward 1	GCGGGCAGGGCGGCCGGCGAACGTAGCGTATAAACTCCG
		Forward 2	GCGGGCCGGCGAACGTAGCGTATAAACTCCC
		Reverse	CGCCAACTTCACCAAGTACTTCACC
Contig114.0547	14	Forward	GCCAATGTTTCCAAGGCTTCATGTT
		Reverse 1	GCGGGCAGGGCGGCGAGGAGCTCTTGGTCCTCCCTTGTC
		Reverse 2	GCGGGCGAGGAGCTCTTGGTCCTCCCTTTTT
Contig136.0560	14	Forward 1	GCGGGCAGGGCGGCCGATCGTTCCGTTTCTGCAAGC
		Forward 2	GCGGGCCGATCGTTCCGTTTCTGCAAGG
		Reverse	ATTCCGACTTCATTGCAGGGCA
Contig539.0530	14	Forward	GGTCTGAATAACGTTGTGTCGAAAA
		Reverse 1	GCGGGCAGGGCGGCAGATGCTGAGAAAACCTTGTGCAAG
		Reverse 2	GCGGGCAGATGCTGAGAAAACCTTGTGCCAA
Contig13.0186	15	Forward 1	GCGGGCAGGGCGGCCGGGAGAAGATTCTTAAGGACACG
		Forward 2	GCGGGCCGGGAGAAGATTCTTAAGGACCCA
		Reverse	CGAACGATACAAAGGCAGCTGGAA
Contig6233.0433	15	Forward	CCTCGCACTCCAACCTGCTTTACAT
		Reverse 1	GCGGGCAGGGCGGCGTCGAAGGAAAACGAAAACGTCGTC
		Reverse 2	GCGGGCGTCGAAGGAAAACGAAAACGTCGTG

Supplemental Table 4. Primers used for PCR amplification and DNA sequencing of *IhCO*

Primer name	Sequences (5´ to 3´)	Fwd / Rev	Position[a]
InCO_pro2F	ACGGTTGTAGCAGTGCAGAACGAAAGAA	Forward	7,614,486 - 7,614,513
InCO_pro4F	CTTGTGGCTGGCTGTGATTCTG	Reverse	7,616,393 - 7,616,414
InCO_proQ65F	GATGCAATTGAGTTGATTAGCC	Forward	–
InCO_proQ65_5F	CGTTGGACGAAGAAGCCCT	Forward	–
InCO_proQ65R	CGAAGTGTGAGTCCTAGTATGAACC	Reverse	–
InCOpro3R	AAGGTAGGGGCAGTCATAGC	Reverse	–
InCO_midF	AAGCCATTGACAGGACAGGT	Forward	7,616,327 - 7,616,346
InCO_pro2R	ATCTTGTGGCTGGCTGTGATTCTGGTGG	Reverse	7,616,389 - 7,616,416
InCO_TerF	CATGATGGTTGGGCTGACGG	Forward	7,617,026 - 7,617,045
InCO_midR	GCATCCTGAGTCAAGAACCC	Reverse	7,617,069 - 7,617,088
InCO_Ter2F	AGTCCTGTTTCTCCTTCGGG	Forward	7,617,673 - 7,617,692
PnCO-realR	CTTTCGAGGCTCTTGAGTGG	Reverse	7,617,855 - 7,617,874
InCO_Ter2R	CGATAGCTGGAATAGTTAATGGGC	Reverse	7,618,263 - 7,618,286

[a] Positions in the *I. nil* genome (Asagao 1.2).
